# Chromosomal Location of *HCA1* and *HCA2*, Hybrid Chlorosis Genes in Rice

**DOI:** 10.1155/2012/649081

**Published:** 2012-02-12

**Authors:** Katsuyuki Ichitani, Yuma Takemoto, Kotaro Iiyama, Satoru Taura, Muneharu Sato

**Affiliations:** ^1^Faculty of Agriculture, Kagoshima University, 1-21-24 Korimoto, Kagoshima, Kagoshima 890-0065, Japan; ^2^Institute of Gene Research, Kagoshima University, 1-21-24 Korimoto, Kagoshima, Kagoshima 890-0065, Japan

## Abstract

Many postzygotic reproductive barrier forms have been reported in plants: hybrid weakness, hybrid necrosis, and hybrid chlorosis. In this study, linkage analysis of the genes causing hybrid chlorosis in F_2_ generation in rice, *HCA1* and *HCA2*, was performed. *HCA1* and *HCA2* are located respectively on the distal regions of the short arms of chromosomes 12 and 11. These regions are known to be highly conserved as a duplicated chromosomal segment. The molecular mechanism causing F_2_ chlorosis deduced from the location of the two genes was discussed. The possibility of the introgression of the chromosomal segments encompassing *HCA1* and/or *HCA2* was also discussed from the viewpoint of Indica-Japonica differentiation.

## 1. Introduction

Many post-zygotic reproductive barrier forms have been reported in plants [1]: hybrid weakness, hybrid necrosis, and hybrid chlorosis. The latter has been observed often in the F_1_ generation from crosses among wheat (*Triticum aestivum* L.) and its relatives [2–6]. This phenomenon resulted from the complementary action of a pair of dominant genes. Research for distribution of these genes contributed greatly to the study of the origin of wheat.

**Figure 1:**
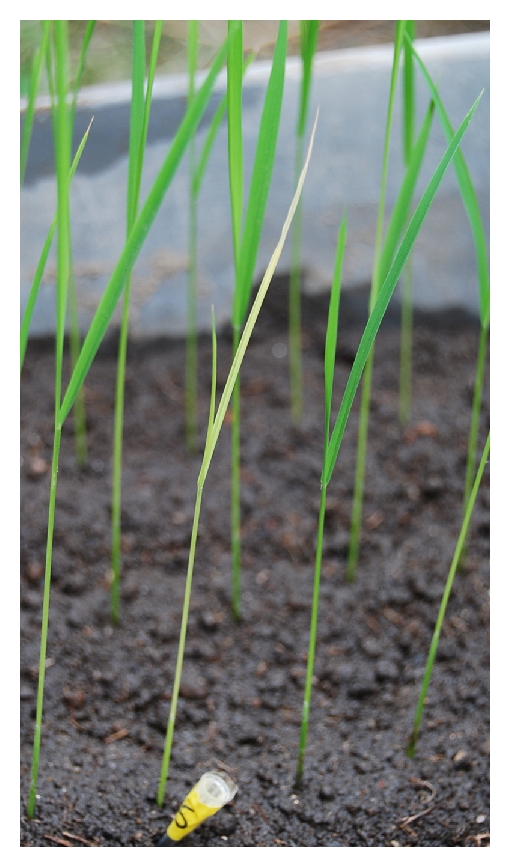
Hybrid chlorosis caused by *hca1-1* and *hca2-1*. Seedlings in an F_4_ line from the cross between J-147 and IR24 are shown 10 days after sowing date. A chlorotic plant is located at the center. The neighboring green plants are normal.

 Hybrid chlorosis in F_2_ generation has been reported only in rice (*Oryza sativa* L.) [7] and interspecific crosses among *Melilotus* species [8]. Sato et al. [7] incidentally found a case of hybrid chlorosis in the F_2_ population from a cross between two Japanese native cultivars: J-147 and J-321. Its first symptom was discoloration of the second or third leaf (Figure 1). The yellowish part expanded gradually. Then the whole plant died within 20 days [9], yielding no seed. The phenomenon was caused by a set of mutually independent duplicated recessive genes, named *hca-1* and *hca-2* by Sato and Morishima [9]. According to the new gene nomenclature system for rice [10], we changed our description of the gene symbols, as shown in Table 1.

Rice is classified into two types: Indica-type and Japonica-type. Sato and Morishima [9] examined the distribution of *HCA1* and *HCA2.* The experimentally obtained results can be summarized as follows. (1) The *hca2-1* gene is widely distributed in native Japonica-type cultivars, whereas many Indica-type cultivars carry its dominant allele, *Hca2-2*. (2) J-147 carries *hca1-1*. This gene is probably rare because the occurrence of F_2_ chlorosis has not been reported in crosses between Taichung 65, which carries *hca2-1*, and many cultivars except for J-147. The mode of inheritance differs between wheat hybrid chlorosis and that of rice, but the distribution of causal genes is related to varietal differentiation in both cases.

 We are interested in genes conferring the post-zygotic reproductive barrier in rice, and we have mapped these genes in the rice genome with the aid of DNA markers [11–14]. We produced hybrids from crosses between J-147 and several cultivars to verify the results of Sato and Morishima [9]. From them, we incidentally found chlorotic plants in the F_2_ population from the cross between J-147 and a Philippine Indica-type cultivar IR24. We have never seen chlorotic plants in F_2_ population from the cross between IR24 and rice cultivars except J-147. Moreover, no reports in the literature describe chlorotic plants in an F_2_ population from the cross with IR24. These facts indicate that IR24 carries *hca2-1*. In general, much DNA polymorphism exists between Japonica-type and Indica-type cultivars. This report describes linkage analysis of *HCA1* and *HCA2* using progeny from the F_2_ population between J-147 and IR24 assisted by DNA markers. Then the molecular mechanism causing F_2_ chlorosis, as deduced from the location of the two genes, is discussed.

## 2. Materials and Methods

### 2.1. Plant Materials

Five rice cultivars were used for this study: J-147, Akihikari, Asominori, Milyang 23, and IR24. Dr. Sato of the Research Institute for Humanity and Nature provided J-147. Dr. Atsushi Yoshimura of Kyushu University provided Asominori and IR24 for this study. Dr. Yoshimichi Fukuta of Japan International Research Center for Agricultural Sciences provided Akihikari and Milyang 23. Akihikari and Milyang 23 are the parents of a set of recombinant inbred (RI) lines developed by Fukuta et al. [15]. Asominori and IR24 are the parents of another set of RI lines developed by Tsunematsu et al. [16]. No report in the relevant literature describes the appearance of chlorotic plants in progeny from a cross between Asominori and IR24, or from a cross between Akihikari and Milyang 23, although hybrid breakdown phenomena were reported for the cross between Asominori and IR24 [17, 18], and in the cross between Akihikari and Milyang 23 [19]. J-147, Akihikari, and Asominori are generally categorized as Japonica-type, whereas Milyang 23 and IR24 are generally categorized as Indica-type. J-147 was crossed with four cultivars: Akihikari, Asominori, Milyang 23, and IR24. F_2_ populations from the above cross combinations were grown in nurseries. The plant spacing was 3 × 3 cm.

Chlorotic plants were segregated in the F_2_ population from the cross between J-147 and IR24 (see Section 3). The normal plants were transplanted to a paddy field in the experimental farm of Kagoshima University to harvest self-pollinated seeds. Approximately 80 plants in each of 16 F_3_ lines were grown in the nursery for the segregation of chlorotic plants. Among them, normal plants in the F_3_ lines in which chlorotic plants segregated were transplanted in the same way as the F_2_ generation. Approximately 80 plants in each F_4_ line were also grown in the nursery for segregation of the chlorotic plants.

### 2.2. Linkage Analysis of HCA1 and HCA2

F_4_ lines in which only *HCA1* or *HCA2* gene was expected to segregate were subjected to linkage analysis using DNA markers. Preliminary analysis using a small number of plants detected the approximate locations of *HCA1* and *HCA2*. Then, we selected the F_4_ lines segregating chlorotic plants in which one locus is fixed for a recessive chlorosis-causing allele, whereas the other locus is heterozygous and the heterozygous chromosomal region encompasses the locus that is extended most. These lines were used for construction of linkage map of *HCA1* or *HCA2*. Linkage analysis was conducted using a computer program (MapDisto ver. 1.7; Lorieux [20]). Map distances were estimated using the Kosambi function [21].

After the linkage analysis, the F_2_ populations from the cross between J-147 and IR24 were grown again. DNA was extracted from each plant and subject to genotyping of the most closely linked DNA markers with *HCA1* and *HCA2* loci to verify that *hca1-1* and *hca2-1* were sufficient to cause hybrid chlorosis in the F_2_ population from the cross between J-147 and IR24.

**Figure 2:**
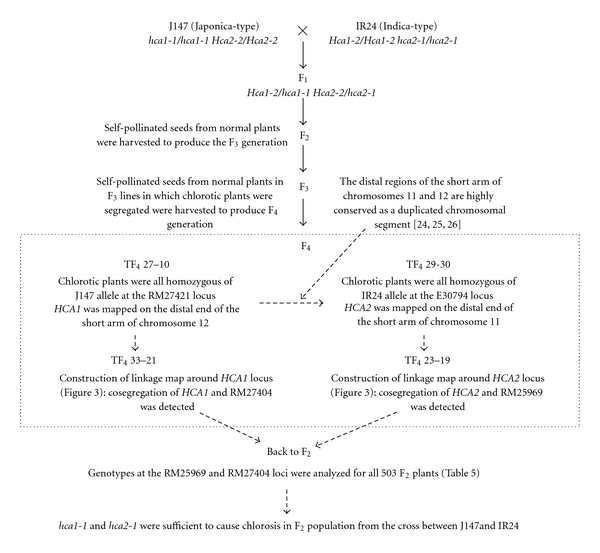
Flow chart showing the breeding of the plant materials for mapping *HCA1* and *HCA2* and for verifying that *hca1-1 *and *hca2-1 *were sufficient to cause hybrid chlorosis in the F_2_ population from the cross between J147 and IR24. Arrows with a solid line indicate the flow of generation of plant materials. Arrows with a dotted line indicate the flow of information on linkage between chlorosis genes and DNA markers.

Figure 2 shows a flow chart depicting the breeding of the plant materials for mapping *HCA1* and *HCA2* and for verification that *hca1-1 *and *hca2-1 *were sufficient to cause hybrid chlorosis in the F_2_ population from the cross between J-147 and IR24.

### 2.3. DNA Marker Analysis

The DNA of plant materials except for the F_2_ population was extracted using the process explained by Dellaporta et al. [22] with some modifications. The DNA of the F_2_ population was extracted according to the experimental protocols of the Rice Genome Project (RGP) (http://rgp.dna.affrc.go.jp/E/rgp/protocols/index.html, written in Japanese) with some modifications [14]. The PCR conditions for indel and SSR markers used for this study were 95°C for 10 min, 40 cycles of 94°C for 30 s, 55°C for 30 s, and 72°C for 30 s with subsequent final extension of 72°C for 1 min. The PCR mixture (5 *μ*L) contained 1 *μ*l of template DNA, 200 mM of each dNTP, 0.2 *μ*M of primers, 0.25 units of Taq polymerase (AmpliTaq Gold; Applied BioSystems), and 1 × buffer containing MgCl_2_. The PCR products were analyzed using electrophoresis in 10% (29 : 1) polyacrylamide gel with subsequent ethidium bromide staining. Then they were viewed under ultraviolet light irradiation. Most PCR-based DNA markers used for this study have already been published. Some primer pairs did not perform well. Therefore, we redesigned them. Details of PCR-based DNA marker design were reported in our previous papers [13, 14].

## 3. Results

The F_2_ populations of the four cultivars with J-147 all produced both normal plants and chlorotic plants (Table 2). The ratios of normal plants and chlorotic plants were fitted to 15 : 1, the expected ratio deduced from the segregation of two independent recessive genes. These results indicated that J-147 carries *hca1-1* gene and that Akihikari, Asominori, Milyang 23, and IR24 carry the *hca2-1* gene.

 Indica-type cultivars and Japonica-type cultivars have frequently shown DNA polymorphism between them. This is true for Asominori and IR24, and for Akihikari and Milyang 23. Using the DNA polymorphism between the two pair of cultivars, DNA marker-based linkage maps were constructed [15, 16]. Results of our recent study indicated much polymorphism between Indica-type cultivars and J-147, and little polymorphism between Japonica-type cultivars and J-147 [14]. Therefore, the progeny from the cross between Indica-type cultivars and J-147 were more suitable for mapping genes. We selected the progeny from the cross between IR24 and J-147 as a mapping population for *HCA1* and *HCA2* because the linkage map constructed from the cross between Asominori and IR24 covered almost the whole genome, whereas that constructed from Akihikari and Milyang 23 had some large gaps, suggesting that some chromosomal regions exist with no DNA polymorphism.

Seeds of normal plants in the F_2_ population were harvested to produce the F_3_ generation. The segregation of chlorotic plants was examined for 16 F_3_ lines from each F_2_ plant. Only normal plants appeared in 10 lines. Chlorotic plants segregated in six lines. In the six lines, the ratios of normal plants: chlorotic plants were all fitted to 15 : 1. In the F_3_ generation, the expected ratio of lines fixed for normal plants, those showing 3 normal: 1 chlorotic segregation, and others showing a 15 normal: 1 chlorotic segregation, was 7 : 4 : 4. However, no lines showing 3 normal: 1 chlorotic segregation appeared. Then, six F_3_ lines in which segregated chlorotic plants were transplanted to a paddy field, and the seeds of normal plants were harvested to produce F_4_ generation.

The segregation of chlorotic plants was examined for 211 F_4_ lines from each F_3_ plant. The line named TF_4_ 27-10 was a progeny from an F_3_ line TF_3_ 27. In TF_4_ 27-10, 70 normal plants and 10 chlorotic plants segregated, showing the maximum chlorotic plant ratio among the 10 TF_3_ 27-derived F_4_ lines tested in our first experiment in the F_4_ generation and significantly deviated from 15 : 1 ratio. Another line named TF_4_ 23-5 was the progeny of an F_3_ line TF_3_ 23. In TF_4_ 23-5, 80 normal plants and 7 chlorotic plants segregated, fitted to a 15 : 1 ratio. We performed a preliminary linkage analysis using a bulked DNA composed of 20 normal plants from TF_4_ 27-10 and another bulked DNA composed of 20 normal plants from TF_4_ 23-5, and 39 DNA marker scattered on all the 12 chromosomes. Because of successive self-pollination, the ratio of heterozygous chromosomal region reduced to approximately 0.25 in the F_4_ generation. *HCA1* and/or *HCA2* are expected to be located on heterozygous chromosomal region of the lines in which chlorotic plants segregated. Among the DNA markers, four showed heterozygosity in both lines. Then DNA from six chlorotic plants in both F_4_ lines was analyzed individually. Possible linkage was observed between chlorosis and KGS1739 [14], a DNA marker located on the short arm of chromosome 12: no homozygotes of IR24 allele were detected. This result suggests that *HCA1* was located on the chromosomal region. Then we analyzed 7 chlorotic plant and 54 normal plants in TF_4_ 27-10 line individually. Results showed that the cosegregation of *HCA1* and the more distal marker RM27421 [23] was detected: all 7 chlorotic plants in this line were homozygous for J-147 allele at the RM27421 locus, whereas all 54 normal plants were heterozygous or homozygous for the IR24 allele at the locus.

**Figure 3:**
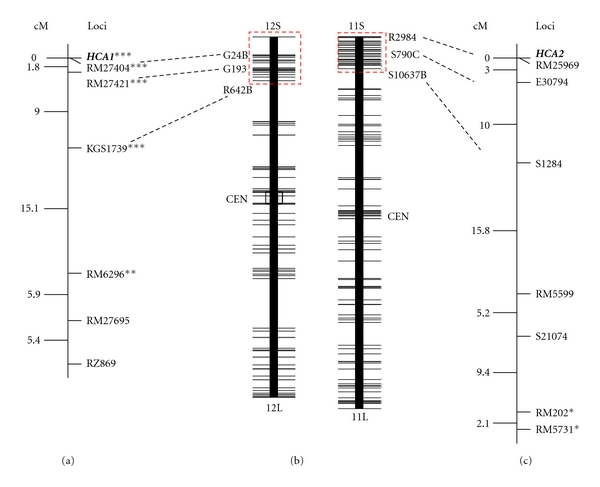
Linkage map showing the respective locations of *HCA1* and* HCA2* on rice chromosomes 12 and 11. (a) Linkage map of *HCA1* on rice chromosome 12 constructed from an F_4_ line derived from the cross between J-147 and IR24 (*n* = 115). (b) RFLP framework maps of rice chromosomes 12 and 11 referred from Harushima et al. [27]. Duplicated genomic regions identified by Wu et al. [26] are enclosed by dotted red squares. (c) Linkage map of *HCA2* on rice chromosome 11constructed from another F_4_ line derived from the cross between J-147 and IR24 (*n* = 119). DNA markers which are located near each other on Nipponbare pseudomolecules are connected with dotted lines. *, **, and *** respectively denote that the gene segregation was significantly deviated from the expected ratio (1 : 2 : 1 or 3 : 1) at 0.05, 0.01, and 0.001 levels.

Based on RFLP-based linkage analysis using the F_2_ population from the cross between Nipponbare and Kasalath, Nagamura et al. [24] reported that the distal regions of the short arms of rice chromosomes 11 and 12 are highly conserved as a duplicated chromosomal segment. Antonio et al. [25] confirmed the high degree of conservation of duplicated segments in these regions using four other mapping populations. Moreover, Wu et al. [26] generated physical maps covering most of the duplicated regions. RM27421 was within the duplicated region on chromosome 12 (Figure 3). These experimentally obtained results and the fact that the hybrid chlorosis was controlled by a couple of recessive duplicate genes led us to the idea that *HCA2* might be located on the distal region of the short arm of chromosome 11. Our preliminary analysis using an F_4_ line named TF_4_ 29-30 showed the cosegregation of *HCA2 *and E30794, an STS marker designed by RGP (http://rgp.dna.affrc.go.jp/E/publicdata/caps/index.html), located on the distal region of the short arm of chromosome 11: all 20 chlorotic plants in this line were homozygous for IR24 allele at the E30794 locus, whereas all 68 normal plants were heterozygous or homozygous for J-147 allele at the locus.

To construct linkage maps around the *HCA1* and *HCA2* loci, we selected F_4_ lines in which one locus is fixed for a recessive chlorosis-causing allele and the other locus is on the heterozygous region extending farthest. Using these lines (*HCA1*: TF_4_ 33-21, *HCA2*: TF_4_ 23-19, see Figure 2), we conducted linkage analysis of each gene using the DNA markers shown in Table 3. The DNA marker sources were the International Rice Genome Sequencing Project [23], RGP, McCouch et al. [28], Chen et al. [29], Monna et al. [30], and Ichitani et al. [14]. The linkage analysis of *HCA1* gene and six DNA markers using 13 chlorotic plants and 102 normal plants in TF_4_ 33-21 showed that *HCA1* was located at the distal end of the short arm of chromosome 12 and that it cosegregated with RM27404 (Figure 3): all 13 chlorotic plants in this line were homozygous for J-147 allele at the RM27404 locus, whereas all 102 normal plants were heterozygous or homozygous for IR24 allele at the locus. The ratio of 102 : 13 did not fit to the expected ratio 3 : 1. Cosegregation of *HCA1* and RM27404 and distorted segregation of the linked DNA markers (Table 4) showed that the distorted segregation of *HCA1* resulted from gametophytic reproductive barrier gene(s), often found in the cross between Indica-type and Japonica-type (see Section 4).

The linkage analysis of *HCA2* gene and seven DNA markers using 23 chlorotic plants and 96 normal plants in TF_4_ 23-19 showed that *HCA2* was located at the distal end of the short arm of chromosome 11 and that it cosegregated with RM25969 (Figure 3): all 23 chlorotic plants in this line were homozygous for IR24 allele at the RM25969 locus, whereas 96 normal plants were heterozygous or homozygous for J-147 allele at the locus. The ratio of 23 chlorotic plants: 96 normal plants fitted the expected ratio 1 : 3. The segregation of the tightly linked DNA markers of *HCA2* also fitted the expected ratio 1 : 2 : 1 (Table 4). RM202 and RM5731, showing distorted segregation, were inherited independently of *HCA2*. Harushima et al. [27] constructed an often-cited restriction fragment length polymorphism (RFLP) marker-based high-density linkage map for rice, in which some RFLP markers have been sequenced. Based on the Nipponbare genome sequence, the relation between our linkage map and that by Harushima et al. [27] is shown in Figure 3.

Using F_4_ generation, we located *HCA1* and *HCA2* genes on the rice genome. However, the ratio of normal plants to chlorotic plants was often distorted from the expected one; that is, the chlorotic plants were often significantly fewer than expected. Therefore, we again produced the F_2_ generation from the cross between J-147 and IR24 to confirm that *hca1-1* and *hca2-1* are sufficient to cause chlorosis in F_2_ generation with the aid of tightly linked DNA markers.

A total of 503 F_2_ plants were classified into 481 normal plants and 22 chlorotic plants. The ratio 481 : 22 looked slightly skewed towards normal plants but fitted the expected ratio 15 : 1 (*χ* ^2^ = 3.033, *P* = 0.08). Genotypes at the RM25969 and RM27404 loci were analyzed for all F_2_ plants (Table 5). No homozygotes of J-147 allele at the RM27404 locus and IR24 allele at the RM25969 locus were present in normal plants. In contrast, all chlorotic plants were homozygotes of J-147 allele at RM27404 locus and IR24 allele at the RM25969 locus. This result indicated that *hca1-1* and *hca2-1* were sufficient to cause chlorosis in F_2_ population from the cross between J-147 and IR24. Segregation of RM25969 fitted the expected ratio 1 : 2 : 1, although that of RM27404 did not fit the expected ratio: homozygotes of IR24 allele were more numerous than expected, and homozygotes of J-147 allele were much fewer than expected. This result is consistent with that of F_4_ generation. The distorted segregation of chlorotic plants in F_3_ generation was thought to result from abnormal segregation at the chromosomal region around the *HCA1* locus.

## 4. Discussion

In this study, *HCA1* and *HCA2* were located on the respective distal regions of the short arms of chromosomes 12 and 11. These regions are known to be highly conserved as a duplicated chromosomal segment [24–26]. According to the Rice Genome Annotation Project (http://rice.plantbiology.msu.edu/index.shtml) [31], 30 genes with known functions are located in common in the areas of interest of chromosomes 11 and 12. Therefore, *HCA1* and *HCA2* are thought to be mutually homoeologous genes, and the loss-of-function mutations of these genes are thought to be the cause of hybrid chlorosis. Based on the symptom, the causal genes of hybrid chlorosis should be involved in chlorophyll synthesis or chloroplast metabolism.

 Recently, two papers reported the reciprocal disruption of duplicate genes causing reproductive barrier in interspecific and intraspecific rice crosses. Yamagata et al. [32] reported that the reciprocal loss of duplicated gene encoding mitochondrial ribosomal protein L27 causes hybrid pollen sterility in F_1_ hybrids of *O. sativa* and *O. glumaepatula*. Mizuta et al. [33] reported disruption of duplicated genes *DOPPELGANGER1* (*DPL1*) and *DOPPELGANGER2* (*DPL2*) causing pollen sterility: independent disruption of *DPL1* and *DPL2* occurred, respectively, in Indica-type and Japonica-type. DPLs encode highly conserved, plant-specific small proteins. In *Arabidopsis thaliana*, recessive embryo lethality is caused by a disruption of duplicated histidinol-phosphate amino-transferase genes encoding for a protein that catalyzes an important pathway leading to histidine incorporated into proteins [34]. The hybrid chlorosis described in this study might offer a new example of a reproductive barrier caused by disruption of duplicate genes.

The segregation of *HCA2* and its tightly linked DNA marker RM27404 was significantly distorted from the expected ratio: the frequencies of Japonica-type alleles were smaller than expected. The segregation distortion of the distal end of the short arm of chromosome 12 was reported in the populations derived from the crosses between Indica-type cultivars and Japonica-type cultivars: IR24 and Asominori [16], and Milyang 23 and Akihikari [35]. The peak of distortion was at an RFLP marker *XNpb193* (= G193), and the frequencies of Japonica-type alleles were smaller than expected in both crosses. Therefore, the segregation distortion observed in this study might result from the same genetic factor(s).

Haplotype analysis around reproductive barrier genes might shed new light on varietal differentiation. Kuboyama et al. [13] performed haplotype analysis around the *HWC2* locus. Carriers of the weakness-inducing allele *Hwc2-1*, most of which are categorized as temperate Japonica-type, share the same haplotype in the 200 kb region between the two DNA markers, KGC4M5 and KGC4M52. The carriers of *hwc2-2* have different haplotypes, most of which are distinct from those of *Hwc2-1* carriers. These results suggested that *Hwc2-1 *diffused in temperate Japonica-types, dragging adjacent genes with it. The* hca2-1 *gene is mainly carried by Japonica-type cultivars. Therefore, the haplotype analysis of *HCA2* might engender new findings related to Japonica-Indica differentiation. We are undertaking haplotype analysis of *HCA2* using a core collection of world rice [36] and a minicollection of Japanese rice landrace [37].

Before the experiment, we expected that Akihikari and Asominori, both generally classified as Japonica-type, carry *hca2-1* gene whereas Milyang 23 and IR24, both generally classified as Indica-type, carry the wild type *Hca2-2*. Based on that expectation, we produced an experimental design in which J-147 was crossed with a set of RI lines so that linkage between *HCA2* and DNA markers was detectable by combining the genotype of each RI line of DNA markers on the whole genome, which had been analyzed by the breeders of the RI lines, and the segregation of chlorotic plants in the F_2_ population between J-147 and each RI line. The experimental design using RI lines for mapping a reproductive-barrier-related gene was successful in a hybrid weakness gene *HWC2 *[11]. However, contrary to our expectation, all four cultivars carry *hca2-1* gene. Milyang 23 is a descendant of IR24. Therefore, *hca2-1* gene of Milyang 23 might derive from IR24. Zhao et al. [38] used a 1,536 SNP panel genotyped across 395 rice diverse accessions to study genomic-wide pattern of polymorphism, to characterize population structure, and to infer the introgression history, revealing that most accessions exhibit some degree of admixture, with many individuals within a population sharing the same introgressed segment because of artificial selection. Therefore, a high probability exists that the *HCA2* locus and its surrounding chromosomal region of IR24 were introgressed from a Japonica-type cultivar. However, another possibility exists: loss-of-function mutation occurred at the *HCA2* locus independently in Indica-type cultivars. The origin of *Hca2-2* allele of J-147 is also interesting. Ichitani et al. [14] reported that J-147 shares the same banding patterns of 38 out of 39 PCR-based DNA markers scattered on the whole genome with the three cultivars generally classified as temperate Japonica-type. This result indicates that J-147 can be categorized as temperate Japonica-type. A DNA marker S1284 (Table 3, Figure 3), located at 2.2 Mbp from distal end of short arm of chromosome 11 (IRGSP pseudomolecules Build05), clearly discriminates Japonica-type and Indica-type, and J-147 carries the Japonica-type allele [14]. The *Hca2-2* allele of J-147 might also be the result of the introgression of small chromosomal segment of the distal end of the short arm of chromosome 11.

To identify the causal genes, we are undertaking closer linkage analysis and linkage disequilibrium analysis of both *HCA1* and *HCA2* genes. Identification of causal genes will contribute to the study of rice varietal differentiation, gene duplication, and chlorophyll synthesis or chloroplast metabolism.

## Figures and Tables

**Table 1:** Gene symbols frequently used in this study according to the new gene nomenclature system for rice [10].

	Gene symbol			
	Sato and Morishima (1988) [9]	This study	Gene full name	Cultivars harboring chlorosis-causing gene
Locus/gene	*hca-1*	*HCA1*	*HYBRID CHLOROSIS A1*	
Recessive allele	*hca-1*	*hca1-1*	*hybrid chlorosis a1-1*	J-147
Dominant allele	*hca-1^+^*	*Hca1-2*	*Hybrid chlorosis a1-2*	

Locus/gene	*hca-2*	*HCA2*	*HYBRID CHLOROSIS A2*	
Recessive allele	*hca-2*	*hca2-1*	*hybrid chlorosis a2-1*	Akihikari, Asominori
				IR24, Milyang 23
				Many Japonica-type cultivars [9]
Dominant allele	*hca-2^+^*	*Hca2-2*	*Hybrid chlorosis a2-2*	

**Table 2:** Segregation for normal and chlorotic plants in the four F_2_ populations.

Cross combination	No. of plants			*χ* ^2^ (15 : 1)	*P*
	Normal	Chlorotic	Total		
J-147 × Milyang 23	56	1	57	1.966	0.161
J-147 × Akihikari	59	1	60	2.151	0.142
J-147 × IR24	62	5	67	0.168	0.682
J-147 × Asominori	60	2	62	0.968	0.325

**Table 3:** Primer sequences designed and used for mapping *HCA1 *and *HCA2* loci.

Marker name	Kind of DNA marker		Primer sequences (5′–3′)	Location on IRGSP pseudomolecules Build05			
				Chromosome	Position		Source
					From	To	
RM25969	SSR	F	TAAATTTGGTTGTCTACGCATGG	11	364229	364418	[23]
		R	CTGCTCCAGATTAGGAGCCAG				redesigned in this study
							
E30794	Indel	F	TCTGCCTATGTATTTGTGCGTTAAT	11	679545	679744	RGP
		R	AAGTAACACAACGAAGGAGCAAC				redesigned in this study
							
S1284	Indel	F	ACATTCAACTGATCACAGCC	11	2186184	2186392	RGP
		R	AGCTCTGCACTAGGATGATG				
							
RM5599	SSR	F	AATTTTGTGCTGTTGTTGAA	11	3810354	3810494	[28]
		R	CTCACAATATCACCATCCAC				
							
S21074	Indel	F	TGCTATAGGTGGTGGTATGC	11	5588871	5589049	RGP
		R	TTTCAAGCTGACGAACGATG				
							
RM202	SSR	F	CCAGCAAGCATGTCAATGTA	11	9050541	9050718	[29]
		R	CAGATTGGAGATGAAGTCCTCC				
							
RM5731	SSR	F	CCTCCACCCTAAGCTTCTCC	11	10005222	10005423	[28]
		R	CGATGCACCTAGCGCATC				
							
RM27404	SSR	F	GCAGCGATTGGAGGTGGAA	12	204766	204863	[23]
		R	GACCGTGCCATCTTGTCCAG				redesigned in this study
							
RM27421	SSR	F	TCAACTCCATTCTACTCTCTACCC	12	468463	468538	[23]
		R	GCTGCTGGTACTTCGTAGAGG				
							
KGS1739	Indel	F	AGAGACGCAGGAGCTGCTTA	12	1999528	1999818	[14, 30]
		R	CATGACCCTTCTATGGCAATTAT				
							
RM6296	SSR	F	CCCACGTTTCTCTTGTCCTC	12	3200580	3200734	[28]
		R	TCTTGCCTCGCTAGGGTTAG				
							
RM27695	SSR	F	CTATAAAGAGTCCGAGGGGTATTGT	12	4828865	4829000	[23]
		R	GGGAGAGGATGTGAATGAAGG				redesigned in this study
							
RZ869	Indel	F	TTTTGGTATTTGCTGCATGG	12	7739507	7739753	RGP
		R	TATCAATCACCCCCAACCTC				

**Table 4:** Segregation of DNA markers linked with *HCA1* and *HCA2* in the mapping population of these genes from the cross between J-147 and IR24.

Marker	Genotype^a^			*n*	*χ* ^2^ (1: 2 : 1)	*P*
	J	H	I			
*HCA1 *(chr. 12)						
RM27404	13	59	43	115	15.730	<0.001
RM27421	12	59	44	115	17.887	<0.001
KGS1739	11	61	43	115	18.235	<0.001
RM6296	15	62	38	115	9.904	0.007
RM27695	19	59	37	115	5.713	0.057
RZ869	21	61	33	115	2.930	0.231

* HCA2 *(chr. 11)						
RM25969	31	65	23	119	2.092	0.351
E30794	28	68	23	119	2.849	0.241
S1284	27	68	24	119	2.580	0.275
RM5599	33	61	25	119	1.151	0.562
S21074	36	63	20	119	4.714	0.095
RM202	42	58	19	119	8.966	0.011
RM5731	43	55	21	119	8.815	0.012

^a^I, H, and J respectively denote homozygote for IR24 allele, and heterozygote and homozygote for J-147 allele.

**Table 5:** Segregation of DNA markers linked with *HCA1* and *HCA2* in the F_2_ population (*n* = 503) derived from the cross between J-147 and IR24. Numbers of chlorotic plants are shown in parentheses.

		Genotypes for RM27404^a^ on chr. 12			Total	**χ** ^2^ (1 : 2 : 1)	*P*
		I	H	J			
Genotypes	I	38 (0)	60 (0)	22 (22)	120		
for	H	94 (0)	139 (0)	37 (0)	270	2.917	0.233
RM25969 on chr. 11	J	37 (0)	66 (0)	10 (0)	113		

	Total	169	265	69			
	*χ* ^2^ (1 : 2 : 1)		41.211				
	*P*		<0.0001				

^a^I, H, and J respectively denote homozygote for IR24 allele and heterozygote, and homozygote for J-147 allele.

## Abbreviations

RGP: Rice genome project
PCR: Polymerase chain reaction
SSR: Simple sequence repeat marker
RI: Recombinant inbred
Indel: Insertion/deletion
RFLP: Restriction fragment length polymorphism.
